# Are We Really Getting Conservation So Badly Wrong?

**DOI:** 10.1371/journal.pbio.1001010

**Published:** 2011-01-25

**Authors:** Kathy MacKinnon

**Affiliations:** 1 Cambridge Conservation Science Group, Department of Zoology, Cambridge, United Kingdom; 2 IUCN/World Commission on Protected Areas

**Figure pbio-1001010-g001:**
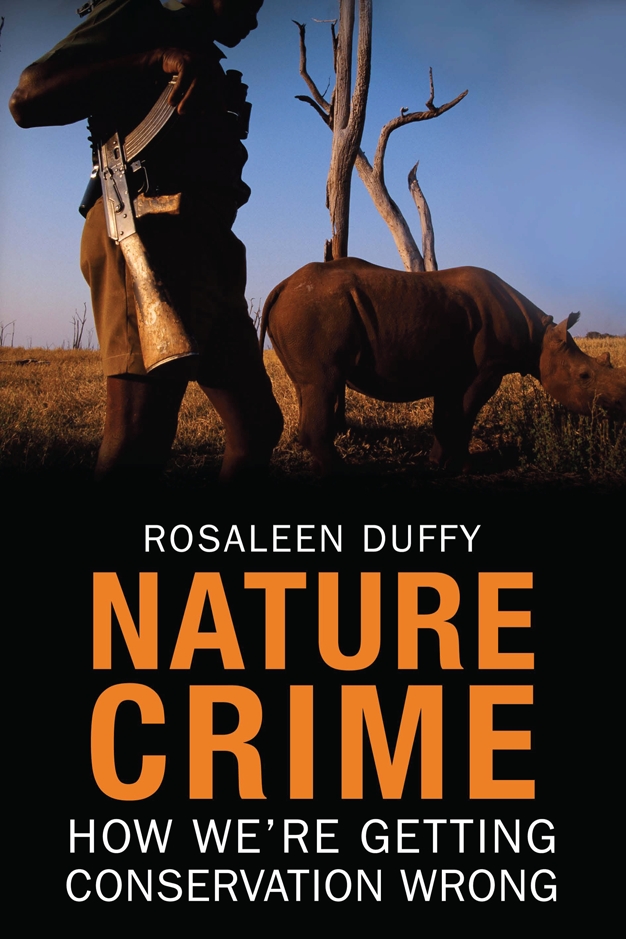
Duffy R (2010) Nature Crime: How We're Getting Conservation Wrong. New Haven, CT: Yale University Press. 288 p. ISBN 978-0300154344 (hardcover). US$42.00.

**About the Author**

**Dr. Kathy Mackinnon** is Vice Chair (Biodiversity) of the IUCN/World Commission on Protected Areas. Until recently she was employed as the Lead Biodiversity Specialist at the World Bank, where she worked on projects to strengthen management of protected areas and to mainstream biodiversity conservation in development programs. She has over 30 years of experience working on conservation projects globally, including 10 years spent in Indonesia on tropical ecology research and working with local and international NGOs. She is the author of over 100 scientific books and publications, including recent books that promote protected areas as proven and sustainable natural solutions helping societies to cope with climate change. In 2007, she was awarded the Distinguished Service Award of the Society of Conservation Biology.

In October 2010, some 18,000 delegates from 193 nations met in Nagoya, Japan, for the 10th Conference of the Parties (COP) to the Convention on Biological Diversity, the international treaty to protect biodiversity. In Nagoya, governments reaffirmed their concern over the continuing loss of biodiversity and set new targets to address the crisis. Among the national delegations, indigenous people, and diverse stakeholders present, lobbying by international nongovernmental organizations (NGOs) contributed to a COP decision to expand protected areas to 17% of terrestrial ecosystems (up from the current 12.9% coverage globally) and marine areas to 10% (currently at just over 1%). Against this background, Rosaleen Duffy argues that mainstream conservation efforts are failing in her new book, *Nature Crime: How We're Getting Conservation Wrong*. Duffy, a professor of international politics at Manchester University, questions the importance of international NGOs in setting global and national conservation agendas, the development of alliances between NGOs and the private sector, and the alienation of local communities by conservation practices (which constitute, in Duffy's words, “the darker side of conservation”).

With chapters that cover the international wildlife trade, global markets, the local costs of conservation, poaching, ivory trade bans, and the role that conflicts play in habitat and resource loss, Duffy addresses many controversial topics in this thought-provoking book. She questions the need for biodiversity conservation to be linked to the concept of wilderness and the exclusion of local people, illustrating her arguments with studies based on her own field work and other publications. The book challenges the idea that poverty is a primary driver of habitat and wildlife loss; many conservationists would agree. The global trade in wildlife, for instance, is big business and so profitable that it is often run by organized crime syndicates that also specialize in drug running and human trafficking. The trade is global, dramatically impacting rare and endangered wildlife in developing countries but also targeting countries such as the United States and United Kingdom as well. It is horrifying to realize that bears are being killed in US national parks so that their gall bladders can be shipped to the Far East for the “traditional medicine trade” and that illegal immigrants lost their lives harvesting cockles in the treacherous sands of Morecombe Bay to provide gourmet dishes for Western Europe.

Because of the influence of global markets on resource use—everything from rhino horn to sapphires and coltan, a metallic ore used in mobile phones—Duffy believes that conservationists, especially the large international NGOs like WWF, Wildlife Conservation Society, Conservation International, and The Nature Conservancy, are taking the wrong approach to saving wild nature. She feels that conservation initiatives focus too little on the real drivers of biodiversity loss—the resource demands of the rich world—and too much on local problems, so that efforts to conserve wildlife criminalize local communities and even promote violence against them. While most conservation funding is targeted to protected areas and supplementing national efforts to protect unique habitats and wildlife, NGOs such as WWF, Conservation International, and Flora and Fauna International are all also involved in awareness campaigns to change resource use patterns in the richer nations. TRAFFIC and Wildlife Conservation Society are working with national agencies to address cross-border illegal wildlife trade. Nevertheless, protected areas are the cornerstones of biodiversity conservation, and for some large-ranging species will be the only places where they can survive. Not all reserves need to be managed by state agencies, however; there is good evidence that reserves managed by indigenous and local communities can be equally or more effective in protecting habitats and species.

Conservationists in general are very aware that establishing new protected areas may reduce access to resources for poor communities. The World Bank and other development agencies even have specific operational policies to mitigate such impacts, and many projects have tried to reconcile the legitimate needs of conservation and local people. Unfortunately, there are no silver bullets in conservation, and most successes involve “trade-offs” and an integrated menu of enforcement, incentives, and champions. Sadly, many integrated conservation and development projects (ICDPs), though well-intentioned, have failed to meet either their conservation or development objectives. New livelihood options tend to be supplementary rather than alternative and often less profitable than the more damaging activities they seek to replace. Why stop illegal logging or clearing protected forest for high-value crops such as cinnamon or coffee if there is no danger of arrest and wealthy and high-placed officials are backing the venture? Moreover, it is totally unrealistic to expect under-resourced conservation organizations to take on responsibility for poverty alleviation and good governance in situations where policy failures, weak government, and poor law enforcement enable illegal logging, wildlife trade, and overexploitation of natural resources. Indeed, the lessons to be drawn from past ICDPs will be highly relevant to implementation of the Reducing Emissions from Deforestation and Degradation (REDD) agenda, which aims to link efforts to reverse climate change with better forest management and conservation.

As human populations continue to grow and natural habitats and species are lost to agricultural expansion and over-harvesting, humankind faces some difficult choices. Biodiversity loss, water shortages, and food security, already serious problems, will become even more urgent environmental issues in the coming decades, and will only worsen with climate change. Protected areas can help to mitigate some of these impacts by storing and sequestering carbon and safeguarding critical ecosystem services—such as water flows and water quality, coastal and flood protection, fisheries production, and pollination—on which all human societies depend. Greater appreciation and protection of these values could help people cope better with the impacts of climate change, especially the poorest and most vulnerable communities for whom Duffy has special concern.

Overall, this book is an interesting, but sometimes infuriating, read. The author raises valid concerns about important issues, but there is much to challenge. For instance, most conservation NGOs (and the World Bank) understand very well the limited potential of ecotourism, which may produce substantial local benefits at some popular sites but is certainly not a universal remedy. While acknowledging the social, economic, and political complexity of many conservation problems, Duffy herself falls into the trap of over-simplification. Her sympathies for local people lead to exaggerated and dramatic statements that conservation promotes “shoot to kill” policies and portrays Africans as “black/bad/poachers/rebels” while conservationists are seen as “white/good/saviors of wildlife.” This may make good copy, but is far from the truth. Conservation is not just a Western agenda. Protected areas are *national* commitments and international and local NGOs work with local field staff. It is hard to overstate the dedication and pride of rangers in the Virunga and Garamba national parks in Democratic Republic of Congo who work to protect endangered wildlife in a region wracked by civil war. Or the empowerment of tribal communities and village women in India who benefit from ecodevelopment projects. From South America to the Pacific islands and from Africa to East Asia, conservation dollars are making a positive difference for wildlife and local peoples. So read the book and think carefully about the arguments. But don't cancel your subscription to WWF just yet.[Fig pbio-1001010-g001]

